# A sprayable Acid-Oxidizing solution containing hypochlorous acid (AOS2020) efficiently and safely inactivates SARS-Cov-2: a new potential solution for upper respiratory tract hygiene

**DOI:** 10.1007/s00405-021-06644-5

**Published:** 2021-02-11

**Authors:** Nadia Giarratana, Balan Rajan, Kannan Kamala, Michelle Mendenhall, Giorgio Reiner

**Affiliations:** 1 APR Applied Pharma Research S.A, 6828 Balerna, Switzerland; 2 Eurofins Advinus Limited, Bengaluru, India; 3 Institute for Antiviral Research, Utah State University, Logan, Utah USA

**Keywords:** Hypochlorous acid (HClO), SARS-CoV-2, Acid-Oxidizing solution (AOS2020), Viral load, Upper respiratory tract

## Abstract

**Introduction:**

To eliminate the COVID-19 pandemic, the transmission of the virus SARS-CoV-2 among the population needs to be blocked and/or at least reduced. Upper respiratory tract viral loads are highest in the early stages of the disease, and high loads are associated with higher mortality rates. This study aims to evaluate the virucidal efficacy of AOS2020, a novel sprayable Acid-Oxidizing solution containing pure and stable hypochlorous acid (HClO), on human coronavirus SARS-Cov-2 in vitro, and the tolerability profile on nasal and oral mucosa suggesting to be a potential solution for upper respiratory hygiene.

**Method:**

Virucidal assays and intranasal and oral irritation tests were undertaken in accordance with relevant national and international guidance and methods.

**Results:**

In pre-clinical tests, the AOS2020, showed > 99.8% virucidal efficacy in < 1 min against SARS-Cov-2. The safety profile testing on both the nasal and oral mucosa indicates that AOS2020 is non-irritant.

**Conclusion:**

These initial results indicate that this product has the potential treatment to reduce viral load in the upper respiratory tract.

## Background

The current COVID-19 pandemic is caused by the SARS-CoV-2, a human coronavirus and is transmitted primarily through [1]:

- Transfer of infectious pathogen saturated respiratory droplets to mucosal surfaces of a recipient, by sneezing, coughing or speaking
- Fomites—contaminated objects such as surfaces and inanimate objects transfer infection when touched

However, those who are infected yet asymptomatic or those who are pre-symptomatic, are potential sources of infection, [2] with modelling suggesting pre-symptomatic transmission of 44%, [3] and real-time data showing 30% [4] and 54% infection rates [5] from such carriers.

Initially, the highest viral loads are found in the upper respiratory tract [6, 7] in diagnosed patients. The World Health Organization (WHO) guidelines recommend covering the nose and mouth, social distancing and good respiratory and hand hygiene [8]. Thus, treatments which can reduce nasal viral load have the potential to minimize the progression and/or spread of the disease. Nasal-spray treatments for respiratory tract viruses have been explored in pre-clinical and other trials [9–11]. A number of potential COVID-19 treatments products delivered by nasal spray have been explored [12, 13]. Hypochlorous acid (HClO) is a potent broad-spectrum fast-acting antibacterial agent with a favourable safety profile [14]. In nasal formulations, it has shown bactericidal, fungicidal, or virucidal effects [15, 16].

A unique Acid-Oxidizing solution (AOS2020) containing pure and stable HClO in a liquid carrier solution [Tehclo Technology™ APR Applied Pharma Research (APR) SA] comprises a hypotonic solution with unique physicochemical characteristics in terms of pH 2.5–3, oxidative reduction potential (ORP) 1000–1200 mV and free chlorine species of which pure HClO is not less than 95%. The solution has been already tested for several toxicological parameters such as cytotoxicity on fibroblasts, phototoxicity, genotoxicity, vaginal, systemic and ocular irritation in acute and chronic (data on file). This study evaluated the virucidal efficacy of AOS2020 on human coronavirus SARS-Cov-2 in vitro and the tolerability profile on nasal and oral mucosa suggesting to be a potential solution for upper respiratory hygiene.

## Methods

### Virucidal assays

The USA_WA1/2020 strain of SARS-CoV-2 was obtained from the World Reference Center for Emerging Viruses and Arboviruses (WRCEVA) at the University of Texas Medical Branch (UTMB, Galveston, TX). A working stock of the virus was prepared by passaging the virus in Vero 76 cells. Test media used was MEM supplemented with 2% FBS and 50 μg/mL gentamicin. The AOS2020 sample was tested at full strength, adding 90% sample to 10% virus solution by volume to achieve a final test concentration of 90% in triplicate. Water was tested in parallel to serve as the virus control. Neutralization and cytotoxicity controls were tested to ensure that virus inactivation did not continue after the specified contact time, and that residual sample in the titer assay plates did not inhibit growth and detection of surviving virus.

The AOS2020 and virus were incubated at room temperature for two contact times of < 1 min or 3 min, followed by 1/10 dilution in test media containing 10% FBS to neutralize. For virus quantification, triplicate neutralized samples from each time point were pooled then serially diluted using eight half-log dilutions in test medium. Each dilution was added to 80–100% confluent Vero 76 cells. On day 6, post-infection plates were scored for presence or absence of viral cytopathic effect (CPE). The Reed–Muench method was used to determine end-point titers (50% cell culture infectious dose, CCID50) of the samples, and the log reduction value (LRV) of the compound compared to the negative (water) control was calculated.

### Intranasal irritation test

This study was performed in an Association for Assessment and Accreditation of Laboratory Animal Care International (AAALAC) accredited laboratory. All procedures were in compliance with the guidelines provided by the Committee for Purpose of Control and Supervision of Experiments on Animals (CPCSEA) Government of India (Registration Number: 2/PO/RcBi/SL/99/CPCSEA) and ISO 10,993-2, Biological evaluation of medical devices—Part 2: Animal welfare requirements. This study plan has been reviewed and approved by the Institutional Animal Ethics Committee (IAEC) of Eurofins Advinus Limited under the Proposal No. 012/Jan-2020 dated 28 January 2020.

The rabbit model has been selected according to the International Standard (ISO 10,993-10) for Medical Device. In addition, the rabbit is often promoted as the prototypical experimental animal as human surrogates in olfaction and inhalation toxicology tests. This is mainly because of the similarities between its sinus anatomy and its immune responses and those of human beings as well as the favourable size of its sinuses and relative ease of access to them [17].

Nine young male New Zealand White Rabbits (not less than 2Kg in weight) have been divided into 3 groups (G1 = control with 0.5 mL saline, G2 = 0.2 mL AOS2020, G3 = 0.5 mL AOS2020 for each nostril) administered twice daily for 5 consecutive days. The decision to consider 5 days of treatment derived from the following considerations and to use the preliminary tolerability profile from rabbit model to support a clinical trial and the final use of the product in humans:

- Once a person is infected exist the 'latent period' before be able to transmit the virus. The current best-estimate of the median latent time is ≈3 days followed by ≈4 days of close to maximal infectiousness [3, 18]. Linton et al. showed a median incubation period of 5.1 days (95% CI 4.5–5.8 days) [19].
- The median time from illness onset to hospital admission was approximately 4 days among cases not known to be deceased at the time of the case report, and 6 days among cases reported as deceased [18].
- According to the standard upper respiratory viruses, if symptoms deteriorate after 5 days of onset or persist beyond 10 days, it is likely that there is secondary bacterial infection. This requires a clinician evaluation [20].

The test item has been intranasally applied using a 1-mL syringe fitted with MAD^®^ (Mucosal Atomization Device) at the interval of 4 h. Local reactions at the site of application were examined twice daily on all application days and evaluated as per the method of Draize [21] Histopathological examination has been carried out on the nasal cavity and evaluation’s scores were recorded as per method B.3 of ISO 10,993-10 and the irritation index is calculated as per method B.4.of ISO 10,993-10. The experiment has been done in a GLP Laboratory.

### Oral irritation test

Toxicon strictly adhered to the United States Department of Agriculture (USDA), Animal and Plant Health Inspection Service, 9CFR, Guide for the Care and Use of Laboratory Animals of National Research Council, 1996 (NIH), Office for Laboratory Animal Welfare (OLAW), ISO 10,993-2 and Association for the Assessment and Accreditation of Laboratory Animal Care (AALAC) standards in maintaining the animal care and use program.

Six Golden Syrian Hamster has been divided into 2 groups for the administration of 0.5 mL of the test item (AOS2020) and 0.5 mL of control (sterile water for injection) for 5 min repeated each hour for 4 h. Macroscopic (immediately after each dose and 24 h after the last dosing) and microscopically (with Irritation Index) evaluation has been done.

## Results

### Effect of AOS2020 on human coronavirus SARS-Cov2

Virus titers and log reduction value (LRV) of SARS-CoV-2 following contact with AOS2020 are shown in Table 1.Virucidal activity was exhibited when the solution was tested at 90% for < 1 min and 3 min, reducing virus from 3.5 log CCID50 per 0.1 mL in virus controls to below the limit of detection of 0.7 logs (> 99.8%).

**Table 1:** Virucidal efficacy of AOS2020 against SARS-CoV-2 after incubation with virus at 22 ± 2 °C

	Concentration	Contact time	Virus titer^a^	LRV^b^
AOS2020	90%	< 1 min	< 0.7	> 2.8
Virus control	n/a	< 1 min	3.5	–
AOS 2020	90%	3 min	< 0.7	> 2.8
Virus control	n/a	3 min	3.5	

^a^Log10 CCID50 of virus per 0.1 mL

^b^LRV (log reduction value) is the reduction of virus compared to the virus control

Neutralization controls demonstrated that residual sample did not inhibit virus growth and detection in the end-point titer assays in wells that did not have cytotoxicity. Positive controls performed as expected, though ethanol was toxic to cells in the 1/10 dilution, limiting the detection of virus to < 1.7 log CCID50 per 0.1 mL.

### Tolerability profile

No clinical signs or pre-terminal deaths were observed in any of the groups and no local reaction was observed during the macroscopic examination at the site of application both at nasal and oral mucosa. The Irritation index has been evaluated on histological samples from oral tissues (the treated cheek pouch) and was determined to be 0.0 indicating that the AOS2020 solution was non-irritant to the buccal tissues of Golden Syrian Hamster.

After 5 consecutive days of nasal treatment, there were no gross lesions in any of the tested animal. The site of application did not reveal any gross findings. The Irritation index on histological samples of nasal mucosa for the 0.2 mL and 0.5 mL dose groups were 0 and 0.083, respectively, indicating that AOS2020 is non-irritant to the nasal mucosa of male New Zealand White Rabbits.

## Discussion

A coronavirus can be inactivated by ultraviolet (UV) light, or heated at 56 °C for 30 min, and is sensitive to disinfectants such as diethyl ether, 75% ethanol, chlorine, peracetic acid, and chloroform [22]. Chlorine-releasing agents act on the capsid and the RNA of several types of viruses [23] and HClO treatments generally render the viral genome irreplicable [24].

The potential effectiveness of free Chlorine species against SARS-CoV-2 for decontamination of equipment and the care environment is acknowledged in guidelines, [25] but no peer-reviewed evidence on the virucidal activity of HClO against SARS-CoV-2 is currently available. However, as it is widely used as a disinfectant in aquatic, food, non-food, and antiseptic applications, the safety of chlorine and chlorine compounds including hypochlorous acid and its salts, has been well documented.

COVID-19 is transmitted predominantly via respiratory droplets [26] with higher viral loads detected in the nose than in the throat, [27] and correlating with disease severity [28]. To monitor disease progression and response to treatment, viral load measurements from tissue samples are routinely used [29]. Zou and colleagues [27] reported that patients with COVID-19 with more severe disease requiring intensive care unit admission, had high viral RNA loads at 10 days and beyond after symptom onset [18]. A recent study indicated that viral loads in severe cases were up to 60 times higher than in mild cases, and a positive association between sputum viral load and disease severity, and the risk of disease progression has been detected in 92 patients with COVID-19 [29].

Starting from the above considerations, these pre-clinical findings suggest that AOS2020 may be effective in treating patients with SARS-Cov-2 infection and by reducing the viral load in the upper respiratory tract to potentially have a role in reducing both the clinical severity of the disease and the spreading of infection. These considerations have to be confirmed with a clinical trial.

It is widely recognized that the simple mechanical instillation of a solution within the nose could reduce the viral/bacterial load [30]. Keeping the nasal cavity clear can enhance the nasal mucosa’s ability to resist the effects of infectious agents, inflammatory mediators and irritants [31]. Ludwig et al. demonstrated that the mechanical activity of cleansing using a solution has an ancillary activity against virusus; the significant reduction of viral load in the nasal wash fluids of patients reduced the duration of disease and recurrence of symptoms [32]. In addition, AOS2020 is a hypotonic solution; considering that the concentration of electrolytes is lower than the average concentration of the mucus due to the osmotic effect, it is absorbed by the mucus and reduces its viscosity (softens/softening the nasal mucus), thus favouring its clearance and removal.

The first antiviral action to block the virus transmission and prevent severe clinical development of the SARS-Cov-2 infection should start from the upper respiratory tract (nose and throat) where the virus starts to replicate into the body. HClO has been shown to exhibit both antibacterial and virucidal activity, and in this study, we have shown that an Acid-Oxidizing solution (AOS2020) containing pure and stable HClO has strong virucidal activity against SARS-Cov-2, with high safety profile on the nasal and oral mucosa. The addition of a strong antiviral efficacy, to the mechanical activity of nasal cleansing, potentially represents an easy and efficient treatment in the panoply of potential COVID-19 treatments.

## Conclusions

This study confirms the virucidal efficacy of AOS2020 against SARS-Cov-2 together with a high tolerability profile on nasal and oral mucosa. As in vitro results, they could be considered as positive indicators of potential activity of AOS2020 for nasal and oral treatment of SARS-Cov-2 infection that should be confirmed with a clinical trial.

## Abbreviations

AOS: Acid-Oxidizing solution containing hypochlorous acid
CCID50: 50% Cell culture infectious dose
COVID-19: Coronavirus disease-2019
CPE: Viral cytopathic effect
HClO: Hypochlorous Acid
MAD^®^: Mucosal atomization device
MERS CoV: Middle east respiratory syndrome coronavirus infection
LRV: Log reduction value
ORP: Oxidative reduction potential
RNA: Ribonucleic acid
SARS CoV2: Severe acute respiratory syndrome coronavirus 2
UV: Ultraviolet light
WHO: World Health Organization
